# Review of Clinical Trials on Effects of Oral Antioxidants on Basic Semen and Other Parameters in Idiopathic Oligoasthenoteratozoospermia

**DOI:** 10.1155/2014/426951

**Published:** 2014-03-31

**Authors:** Senka Imamovic Kumalic, Bojana Pinter

**Affiliations:** Reproductive Unit, Division of Gynecology and Obstetrics, University Medical Centre Ljubljana, Slajmerjeva 3, 1000 Ljubljana, Slovenia

## Abstract

Infertility affects 50 to 80 million people worldwide. Male factor is a cause of infertility in almost half of cases, mainly due to oligoasthenoteratozoospermia (OAT). With common diagnostic methods no cause can be found in approximately 30% of cases of male infertility due to OAT and these are considered idiopathic. Reactive oxygen species (ROS) play an important role in male infertility and are proved to be higher in infertile men; antioxidants could oppose their effect. The aim of this paper was to review the literature on clinical trials in the period from year 2000 to year 2013 studying the effects of various types of antioxidant supplements on basic and other sperm parameters and pregnancy rates in subfertile males with idiopathic OAT. The majority of studies were randomized and placebo controlled and confirmed beneficial effect of antioxidants on at least one of the semen parameters; the biggest effect was determined on sperm motility. In many of these trials combinations of more antioxidants were assessed. The optimal dosages of one or more antioxidants were not defined. We concluded that antioxidants play an important role in protecting semen from ROS and can improve basic sperm parameters in case of idiopathic OAT.

## 1. Introduction

Almost 15% of all couples trying to conceive are affected by infertility, and in almost half of these cases male infertility is the sole or a contributing factor [1]. While conditions such as varicocele, cryptorchidism, and hypogonadism are definable causes for infertility, no cause may be determined for an abnormal semen analysis in over 25% of cases [2]. Such idiopathic infertility and oligoasthenoteratospermia (iOATs) is a condition in which sperm concentration, the proportion of motile sperms, and the proportion of morphologically normal sperms are below the World Health Organization (WHO) reference values [3].

Elevated reactive oxygen species (ROS) levels in the semen may be an etiologic factor for male infertility [4]. It is estimated that 25% of infertile men possess high levels of semen ROS, whereas fertile men do not have high levels of semen ROS [5, 6]. ROS are needed for capacitation, the acrosome reaction, and ultimately fertilization [7]. However, their uncontrolled production is detrimental to cell function as they damage a variety of biomolecules such as lipids, amino acids, carbohydrates, protein, and DNA and adversely affect sperm function [8] due to DNA damage [9, 10], reduced motility [11], and defective membrane integrity [12, 13]. Spermatozoa are particularly susceptible to oxidative injury due to the abundance of plasma membrane polyunsaturated fatty acids. These unsaturated fatty acids provide fluidity that is necessary for membrane fusion events (e.g., the acrosome reaction and sperm-egg interaction) and for sperm motility [14]. The human ejaculate contains a number of potential sources of ROS. These include leukocytes, germ cells, or abnormal sperms [15]. At the same time, a number of cellular molecules called antioxidants, which protect the cell from excessive ROS-induced lipid peroxidation, are also present within the ejaculate [16]. Studies have shown that seminal antioxidant capacity is suppressed in infertile men with high ROS levels compared to men with normal levels of ROS [17, 18].

## 2. Materials and Methods

We searched PubMed with keywords, including combinations of search terms such as “male infertility” and “antioxidants.” We searched for reviews, controlled and randomized controlled clinical studies. From the numerous search results for the period between 1st January 2000 and 31st December 2013, 32 primary studies on idiopathic oligoasthenoteratozoospermia (OAT) were chosen and their data were gathered in order to provide a complete overview of the literature. Given the different antioxidants used (both alone and in combination), the different dosages, different duration of treatment, and various number of participants (from very small groups to large researches), we looked up for statistical significance of changes in basic sperm parameters and pregnancy rates.

## 3. Results and Discussion

The review of the studies on antioxidants in clinical studies is illustrated in Table 1.

### 3.1. Sperm Concentration

Low sperm concentration or oligozoospermia is defined as concentration less than 15 × 10^6^ spermatozoa/mL according to WHO reference value from 2010 [51] and less than 20 × 10^6^ spermatozoa/mL according to WHO reference values from 1999 [52], which were considered in most of researches in this review. Many researches showed significant improvements in sperm concentration after oral intake of different antioxidants [19–31]. Most of these researches investigated combination of different antioxidants, like L-carnitine, coenzyme Q10 (CoQ10), vitamin C, vitamin E, zinc (Zn), selenium (Se), and so forth. But there are also some studies that investigated only one type of antioxidant. Safarinejad et al. showed that intake of 200 mg CoQ10 daily for 26 weeks improved sperm concentration in study group (28.7 ± 4.6 × 10^6^ spermatozoa/mL) versus placebo group (16.8 ± 4.4 × 10^6^ spermatozoa/mL) (*P* = 0.005) [23]. After 6 months of intake of combination of 25 mg clomiphene citrate and 400 mg vitamin E per day sperm concentration improved from 10.2 × 10^6^ ± 4.14 spermatozoa/mL to 18 × 10^6^ ± 15 spermatozoa/mL (*P* = 0.0025) [26]. There was also significant improvement in sperm concentration from 14.3 ± 7.38 × 10^6^ spermatozoa/mL to 32.8 ± 10.3 × 10^6^ spermatozoa/mL (*P* < 0.001) after consumption of 1 g of vitamin C twice daily taken for 2 months as proved by Akmal et al. [28].

### 3.2. Sperm Motility

Asthenozoospermia is defined as less than 40% of motile spermatozoa [51] and according to WHO reference value from 1999 less than 50% of motile spermatozoa [52]. 20 out of 32 studies in our review proved significant improvement in sperm motility after the use of antioxidants [19, 20, 22–39]. Improvement in sperm motility has been shown mostly in researches considering mixture of more antioxidants such as selenium and vitamin E [38, 39]. Most of studies with just one type of antioxidant were about CoQ10 but in different dosages and in different duration of consuming [22–24, 37]. Kumar et al. showed that consumption of herbal-mineral supplement Addyzoa for 3 months improved total and progressive sperm motility in study group. Total motility improved from 23.2 ± 17.3% before the treatment to 33.4 ± 23.2% after the treatment (*P* = 0.008). Progressive motility improved from 15.7 ± 12.6% before treatment to 22.6 ± 18.0% after treatment with Addyzoa (*P* = 0.024) [33]. Wang et al. showed that L-carnitine in combination with vitamin E taken for 3 months significantly improved forward sperm motility from 28.6% ± 9.2% to 45.4% ± 11.1% (*P* < 0.01), compared with just vitamin E [35]. After treatment with 200 mg CoQ10 twice daily for 6 months sperm motility improved from 9.13% ± 2.50% before the therapy to 16.34% ± 3.43% after the therapy (*P* < 0.05) [37].

### 3.3. Sperm Morphology

WHO reference values from 1999 [52] defined teratozoospermia as less than 14% of normal shape and form spermatozoa according to strict Krüger criteria. Although WHO reference values from 2010 define teratozoospermia as less than 4% of normal shape and form spermatozoa [51] strict Krüger criteria are still used as reference value for assessing sperm morphology. L-carnitine in combination with CoQ10, vitamins E and C, zinc, selenium [20, 40], CoQ10 alone [23, 24], pentoxifylline [25], N-acetyl-cysteine with Se [27], vitamin C alone [28], combination of papaya, beta-glucan, lactoferrin, vitamins C and E [36], Se, and vitamin E [38], and pycnogenol [41] significantly improved sperm morphology. Therapy with 200 mg CoQ10 daily for 26 weeks improved sperm morphology in 114 participants in study group to 17.6% ± 4.4% versus 14.8% ± 4.1% in 114 participants in placebo group (*P* = 0.01) [23]. Safarinejad also showed that intake of 400 mg of pentoxifylline twice daily for 24 weeks of treatment phase significantly improved percentage of sperm with normal morphology to 25.4 ± 4.3% in study group versus 17.4 ± 4.2% in placebo group (*P* = 0.001) [25]. Combination of 20 mg beta-glucan, 50 mg fermented papaya, 97 mg lactoferrin, 30 mg vitamin C, and 5 mg vitamin E, twice per day for 3 months, improved percentage of morphologically normal sperm in 36 participants from 17.0 ± 5.2% to 29.8 ± 6.5% (*P* < 0.01) [36].

### 3.4. Sperm DNA Fragmentation and Chromatin Integrity

ROS can cause sperm DNA damage and integrity of sperm DNA can be measured with DNA fragmentation. The levels of sperm-derived ROS (measured in sperm preparations having minimal leukocyte contamination) have been associated with sperm DNA damage [53]. High level of denatured DNA in spermatozoa with large nuclear vacuole could arise from precocious decondensation and disaggregation of sperm chromatin fibers [54]. Dietary antioxidants may be beneficial in reducing sperm DNA damage, particularly, in men with high levels of DNA fragmentation [5]. Five out of 32 studies confirmed that the usage of different antioxidants had important influence on DNA fragmentation and chromatin integrity [20, 42–46]. Song et al. showed that combination of Chinese medicine Compound Xuanju Capsule with vitamin E taken for 3 months decreased degree of DNA fragmentation index (DFI) after therapy to 29.57 ± 12.19 compared just to vitamin E with degree of DFI of 34.09 ± 10.32 (*P* < 0.05) [42]. Greco et al. [44, 45] had proved that 1 g of vitamin C and 1 g of vitamin E together taken for 2 months significantly decreased the degree of DNA fragmentation from 22.1 ± 7.7 to 9.1 ± 7.2 (*P* < 0.001) [45]. Raigani et al. showed that zinc sulphate significantly improved sperm chromatin integrity [46].

### 3.5. Pregnancy Rate

CoQ10 [24], clomiphene citrate with vitamin E [26], lycopene [31], L-carnitine with vitamin E [34, 35], and selenium with vitamin E [38] significantly improved spontaneous pregnancy rates during duration of treatment, while L-carnitine with cinnoxicam [40] and vitamins C and E together [44] significantly improved pregnancy rates per cycle after assisted reproductive technology with intracytoplasmic sperm injection (ICSI). Ghanem et al. proved higher spontaneous pregnancy rate in 30 participants after the intake of combination of 25 mg clomiphene citrate and 400 mg vitamin E per day for 6 months (36.7%) than in placebo group (13.3%) with *P* = 0.037 [26]. L-Carnitine, 2 g, with vitamin E taken for 3 months improved spontaneous pregnancy rate to 31.1% compared to vitamin E group with pregnancy rate of 3.8% (*P* < 0.01) [35]. Another example in study by Greco et al. confirmed higher pregnancy rate after 2 months therapy with 1 g of vitamin C and 1 g of vitamin E daily. After ICSI clinical pregnancy rate was 48.2% after therapy versus 6.9% before therapy (*P* < 0.05) [44].

### 3.6. Negative or No Effect on Sperm Parameters

In this review we find out also rare negative effects of antioxidants on sperm parameters or no effect. Pycnogenol caused nonsignificant fall in baseline sperm count by 10% [41]. Similarly, treatment with vitamins C and E, ß-carotene, zinc, and selenium significantly increased sperm decondensation [43]. Large research on saffron showed no statistically significant improvements in any of the studied semen parameters [48].

### 3.7. Other Parameters

We looked at the basic sperm parameters but there were also many other positive influences; for example, CoQ10 and pentoxifylline caused improvements in total antioxidant capacity and acrosome reaction [22, 25, 49, 50]; FSH value [22, 23] decreased after CoQ10 treatment, semen leucocyte concentration decreased [36], and level of ROS [50] decreased after antioxidant mixtures. Antioxidants protect unsaturated fatty acids and so provide fluidity that is necessary for membrane fusion events like the acrosome reaction. Although hormonal abnormalities are not always evident, iOAT is sometimes associated with lower serum testosterone and inhibin levels and higher serum estradiol, LH, and FSH levels [55, 56]. The increased serum FSH level in men with azoospermia or severe oligozoospermia indicates damaged seminiferous tubule [57] and is inversely associated with sperm concentration, motility, and morphology [58]. ROS has been found in the seminiferous tubules and seminal plasma of most patients with iOAT [59]. Decreased levels of ROS due to antioxidant consumption can cause fall in serum FSH level. Leukocytes are potential source of ROS and due to protective influence of antioxidants their concentration may decrease [15]. In addition, studies have found an increase in inhibin B value [23] and in superoxide-dismutase- (SOD-) like and catalase activity [21, 25], which among others represent the total antioxidant capacity of seminal plasma [60]. Inhibin B in positively correlated with sperm concentration and is, like FSH, thought to be a marker of spermatogenesis and Sertoli cell function [61, 62].

## 4. Conclusions

Most of the published studies were randomized and placebo controlled. The majority of studies confirmed beneficial effect of different antioxidants on at least one of the semen parameters and the biggest effect was determined on sperm motility. In many of these trials combinations of more antioxidants were assessed. The optimal dosages of one or more antioxidants were not defined.

Most commonly antioxidants studied were vitamin E, vitamin C, selenium, CoQ10, N-acetyl-cysteine, L-carnitine, and zinc and their favorable effect was confirmed. According to this review favorable effects on iOAT have been determined with CoQ10, vitamin E, selenium, and also vitamin C and N-acetyl-cysteine treatments. In case of oligozoospermia vitamin E and CoQ10 were most often proved to be effective. Favorable effects on asthenozoospermia have most often been determined with vitamin E, CoQ10, and selenium treatments. In teratozoospermia selenium and CoQ10 treatments were most often proved to be effective. In addition, combination of vitamin C and E showed the biggest favorable effect on DNA fragmentation; similar effects were determined with zinc and selenium treatments.

In conclusion, antioxidants play an important role in protecting semen from ROS and can improve basic sperm parameters in case of idiopathic oligoasthenoteratozoospermia.

## Figures and Tables

**Table 1:** Study characteristics and the effect of oral antioxidants on basic and other semen parameters.

Study/author	Year	Patients/test	Number of patients	Antioxidant/duration of th.	Significant improvement	Nonsignificant improvement	Negative effect
Wirleitner et al. [19]	2012	OAT versus non-OAT, MSOME	147	Fertilovit Mplus/2–12 months	↑ concentration and motility of sperm	Morphology	

Abad et al. [20]	2013	AT/DFI, basic sperm parameters	20	L-Carnitine 1500 mg; vitamin C 60 mg; CoQ10 20 mg; vitamin E 10 mg; Zn 10 mg; vitamin B9 200 *μ*g; Se 50 *μ*g; vitamin B1 21 *μ*g/3 months	DNA integrity (*P* < 0.01), the proportion of DDS ↓ (*P* < 0.05). ↑ in concentration, motility, vitality, and morphology parameters.		

Safarinejad [21]	2011	iOAT	238 (analysis on 211) SG: 106 PG: 105	SG: eicosapentaenoic (EPA) and docosahexaenoic acids (DHA), 1.84 g per day versus PG/32 weeks	SG: ↑ of sperm cell total count (from 38.7 ± 8.7 × 10^6^ to 61.7 ± 11.2 × 10^6^, *P* = 0.001) and sperm cell concentration (from 15.6 ± 4.1 × 10^6^/mL to 28.7 ± 4.4 × 10^6^/mL, *P* = 0.001). Seminal plasma EPA and DHA conc. were positively correlated with seminal plasma SOD-like and catalase-like activity (both *P* = 0.001).	In seminal plasma, both SOD-like and catalase-like activity were positively correlated with sperm count, sperm motility, and sperm morphology.	

Safarinejad [22]	2009	iOAT/semen analyses, AR, immunobead test for antisperm antibody, and determination of resting levels of LH, FSH, prolactin, testosterone, and inhibin B	212 (SG: 106, versus PG: 106)	CoQ10 300 mg/26 weeks followed by a 30-week treatment-free phase	SG: ↑ in sperm density and motility (each *P* = 0.01). ↓ FSH and LH at the 26-week treatment phase (each *P* = 0.03). By the end of the treatment phase the mean AR had increased from 14% ± 8% and 15% ± 8% to 31% ± 11% and 16% ± 10% in the CoQ10 and placebo groups, respectively (*P* = 0.01).		

Safarinejad et al. [23]	2012	iOAT/semen parameters, seminal plasma TAC, FSH, and inhibin B	228 SG: 114 PG: 114	CoQ10 200 mg/day/26 weeks	SG: ↑ in sperm density (28.7 ± 4.6 × 10^6^/mL versus 16.8 ± 4.4 × 10^6^/mL (*P* = 0.005)), sperm motility (35.8% ± 2.7% versus 25.4% ± 2.1% (*P* = 0.008)), and sperm morphology (17.6% ± 4.4% versus 14.8% ± 4.1% (*P* = 0.01)). FSH ↓ (*P* = 0.02), inhibin B ↑ (*P* = 0.01)		

Safarinejad [24]	2012	iOAT/semen parameters and pregnancy rates	287	CoQ10 300 mg orally twice daily/12 months	Mean sperm conc., sperm progressive motility, and sperm with normal morphology improved by 113.7, 104.8, and 78.9%, respectively (all *P* < 0.05).	The overall spontaneous pregnancy rate was 34.1% within a mean of 8.4 ± 4.7 months.	

Safarinejad [25]	2011	iOAT/semen parameters, testosterone, LH, FSH, and inhibin B, seminal plasma SOD-like activity, and acrosome reaction	254 (SG: 127, PG: 127)	SG: PTX (pentoxifylline) 400 mg twice daily/4-week screening phase, a 24-week treatment phase, and a 12-week treatment-free period	SG after PTX: ↑ sperm conc. (mean value, from 26.4 ± 4.6 × 10^6^/mL to 16.2 ± 3.4 × 10^6^/mL), sperm motility (mean value, from 35.8 ± 4.2% to 26.4 ± 2.4%), and sperm with normal morphology (mean value, from 25.4 ± 4.3% to 17.4 ± 4.2%) (all *P* = 0.001); mean SOD-like and catalase-like activity ↑ than in the semen of PG (46.4 ± 2.4 versus 36.3 ± 1.3 U/mL and 371 ± 44 versus 301 ± 14 U/mL, respectively, both *P* = 0.003). The AR was observed to be ↑ in PTX group (*P* = 0.01).		

Ghanem et al. [26]	2010	iOA/basic semen parameters, pregnancy incidence	SG: 30 PG: 30	Clomiphene citrate 25 mg/day + vit. E 400 mg/day/6 months	SG: sperm conc.: 10.2 × 10^6^ ± 4.14 → 18 × 10^6^ ± 15(*P* = 0.0025); progressive motility: 4% ± 6 → 7% ± 10 (*P* = 0.0286). Spontaneous pregnancy incidence, SG: 36.7%,versus PG: 13.3% (*P* = 0.037)		

M. R. Safarinejad and S. Safarinejad [27]	2009	iOAT/serum T estradiol, FSH, LH, prolactin, inhibin B, Se, and N-acetyl-cysteine. Semen analysis, seminal plasma Se, and N-acetyl-cysteine.	468 SG 1: 116 SG 2: 118 SG 3: 116 PG: 118	SG 1: Se 200 *μ*g/day SG 2: N-acetyl-cysteine 600 mg/day SG 3: Se 200 *μ*g + N-acet-cys 600 mg/day/26 weeks + 30-week treatment-free period	A strong correlation was observed between the sum of the Se and N-acetyl-cysteine concentrations, and mean sperm concentration (*r* = 0.67, *P* = 0.01), sperm motility (*r* = 0.64, *P* = 0.01), and percent normal morphology (*r* = 0.66, *P* = 0.01).	Se + N-ac-cy.: ↓ FSH, ↑ T, inhibin B	

Akmal et al. [28]	2006	O/semen parameters	13	vitamin C 1 g twice daily/2 months	Mean sperm count: 14.3 ± 7.38 × 10^6^ sperms/mL to 32.8 ± 10.3 × 10^6^ sperms/mL (*P* < 0.001), mean sperm motility: 31.2 ± 9.61% to 60.1 ± 8.47% (*P* < 0.001), and mean sperms with normal morphology: 43 ± 7.87% to 66.7 ± 4.77% (*P* < 0.001).		

Shi et al. [29]	2004	OA/seminal routine analysis	34	Xinxibao (Zn and Se tablets) three times a day/90 days + five tablets at a time for 90 days in succession	The sperm quality was improved 60 days and 90 days after treatment. 5 cases (14.7%) showed remarkable effect, 25 (73.5%) improved.	4 cases (11.8%) did not respond.	

Suzuki et al. [30]	2003	O and A versus normozoospermia/sperm parameters, serum hormones, and SOD activity in the serum and the seminal plasma + the testicular artery	SG: 47 CG: 16	Sairei-to 9 g/day/3 months	SG: total sperm conc. (17.1 ± 20.0 to 28.7 ± 35.5 × 10^6^/mL, *P* = 0.02) and sperm motility (30.1% ± 21.6 to 45.8% ± 24.4, *P* < 0.0001) and the pulsatility index of the testicular artery ↓ (2.03 ± 0.84 to 1.64 ± 0.48, *P* = 0.04)	After th. serum hormones and SOD activity did not change significantly in either group. CG: no significant change in sperm conditions or testicular artery flow.	

Gupta and Kumar [31]	2002	Idiopathic nonobstructive O/A/T spermia/semen analysis	30	Lycopene 2000 mcg, twice a day/3 months	20 patients (66%): ↑sperm conc., 16 (53%) ↑ motility. The median change in concentration was 22 million/mL, motility 25%. Higher baseline concentrations (more than 5 million/mL) were associated with significant improvement and resulted in six spontaneous pregnancies in 26 patients (23%).	14 patients (46%) ↑ in sperm morphology (median change 10%). Baseline sperm concentration less than 5 million/mL was associated with no significant improvement.	

Busetto et al. [32]	2012	Idiopathic AT/basic sperm parameters	114 (96 finished)	L-Carnitine 145 mg, acetyl-L-carnitine 64 mg, fructose 250 mg, citric acid 50 mg, Se 50 *μ*g, CoQ10 20 mg, Zn 10 mg, ascorbic acid 90 mg, cyanocobalamin 1.5 *μ*g, and folic acid 200 mcg once a day/4 months	↑ Mean sperm progressive motility: 18.3 ± 3.8 to 42.1 ± 5.5, 16 patients achieved pregnancy during the study.	Concentration and morphology	

Kumar et al. [33]	2011	At least one parameter of OAT/basic semen parameters, ROS, TAC, and DFI (SCSA)	SG: 21 PG: 23	herbal-mineral supplement Addyzoa/3 months	SG: total motility: 23.2 ± 17.3% → 33.4 ± 23.2% (*P* = 0.008) Progressive motility: 15.7 ± 12.6% → 22.6 ± 18.0% (*P* = 0.024)		

Chen et al. [34]	2012	O, A/sperm concentration and % of progressively motile sperm, the rate of clinical pregnancy	Oligosp: 64 (SG: 33 + CG: 31) Astheno: 42 (SG: 22 + CG: 20)	Oligospermia: CG: tamoxifen 10 mg bid SG: tamoxifen 10 mg bid + vit. E 100 mg tid Asthenospermia: CG: levocarnitine oral solution 1 bottle bid SG: levocarnitine oral solution 1 bottle bid + vit. E 100 mg tid/3 months	Oligospermia: the number of spontaneous pregnancies after th. were CG: 0, and SG: 6 (*P* < 0.01). Asthenospermia: after th. the numbers of cases evaluated as with no or slight improvement in the % of progressively motile sperm were 7 and 2 (*P* < 0.01), 4 and 8 (*P* < 0.01), and the number of spontaneous pregnancies CG: 5, and SG: 9 (*P* < 0.01).	Asthenosperm: after th, the number of cases evaluated as with moderate or marked improvement in the percentage of progressively motile sperm was 3 and 2 (*P* > 0.05) and 1 and 1 (*P* > 0.05)	

Wang et al. [35]	2010	A/basic sperm parameters	135 Group A (*n* = 68) and B (*n* = 67)	Group A: L-carnitine 2 g/day + vitamin E Group B: vitamin E/3 months	Group A: ↑ % of forward motile sperm (28.6% ± 9.2% to 45.4% ± 11.1%, *P* < 0.01), the rate of spontaneous pregnancy ↑ (31.1%) than in group B (3.8%) after the treatment (*P* < 0.01).	Group A: sperm density and the % of the sperm of normal morphology (*P* > 0.05).	

Piomboni et al. [36]	2008	AT + leukocytosis/sperm parameters, DNA damage (acridine orange)	51 (SG: 36 + CG: 15)	SG: beta-glucan 20 mg, fermented papaya 50 mg, lactoferrin 97 mg, vit. C 30 mg, and vit. E 5 mg, twice per day/3 months	SG: % of morphologically normal sperm (17.0 ± 5.2 to 29.8 ± 6.5) and total progressive motility (19.0 ± 7.8 to 34.8 ± 6.8), ↓ in leukocyte conc. (2.2 ± 0.9 to 0.9 ± 0.2), all *P* < 0.01	Structural sperm characteristics as well as chromatin integrity were also improved after treatment.	

Balercia et al. [37]	2004	iA (WHO 1999)/basic sperm parameters, seminal plasma and sperm CoQ10, and phosphatidylcholine (PC)	22	CoQ10 200 mg 2x/day/6 months	CoQ10 sem. plasma (ng/mL: 42.0 ± 5.1 to 127.1 ± 1.9 (*P* < 0.005)) CoQ10 sperm cells (ng/10^6^ cells): 3.1 ± 0.4 to 6.5 ± 0.3 (*P* < 0.05) PC sem. plasma (*μ*M): 1.49 ± 0.50 to 5.84 ± 1.15 (*P* < 0.05) PC sperm cells (nmol/10^6^ cells): 6.83 ± 0.98 to 9.67 ± 1.23 (*P* < 0.05) Sperm cell motility 9.13 ± 2.50% to 16.34 ± 3.43% after th. (*P* < 0.05)	Sperm conc. and sperm morphology	

Moslemi and Tavanbakhsh [38]	2011	iAT/semen parameters and pregnancy rates	690 (analysis on 525)	Se 200 *μ*g + vitamin E 400 units/min. 100 days	52.6% (362 cases) total improvement in sperm motility, morphology, or both and 10.8% (75 cases) spontaneous pregnancy versus no treatment (95% confidence interval): 3.08 to 5.52; *P* ≤ 0.001	No response to treatment occurred in 253 cases (36.6%)	

Keskes-Ammar et al. [39]	2003	Infertile men/basic sperm parameters, MDA, and serum vitamin E level.	54: SG: 28 (20 analyzed) CG: 26	SG: vitamin E 400 mg + Se 225 *μ*g/day CG: vitamin B 4.5 g/day/3 months	SG: ↓ in MDA concentrations and an ↑ of sperm motility		

Cavallini et al. [40]	2012	Idiopathic OAT/basic sperm parameters and aneuploidy (FISH)	55 (analysis on 33: 22 responder—group 1 + 11 nonresponder—group 2)	L-carnitine 1 g given twice per day + acetyl-L-carnitine 500 mg given twice per day + one 30 mg cinnoxicam tablet every 4 days/3 months	Group 1 versus group 2: improvement in morphology and number of aneuploid spermatozoa (*P* < 0.01); ↑ % of biochemical pregnancy after ICSI (54.4% versus 9.1%, *P* < 0.01), clinical pregnancy after ICSI (50% versus 9.1%, *P* < 0.01), and live births (45.4% versus 9.1%, *P* < 0.01)	Numbers of oocytes fertilized and embryos transferred	

Roseff [41]	2002	Subfertile/basic sperm parameters before and after capacitation and mannose receptor binding	19	Pycnogenol 200 mg daily orally/90 days	The mean sperm morphology following Ham's F-10 capacitation ↑ by 38% following th. (*P* < 0.001) and the mannose receptor binding assay scores improved by 19% (*P* < 0.005)	Baseline morphology ↑ after th. by 33%	The mean % change from baseline sperm count after th ↓ nonsignificantly by 10%

Song et al. [42]	2012	Idiopathic OA/basic sperm parameters, DFI (SCSA)	SG: 24 CG: 26	SG: vit. E + xuanju caps CG: vit. E/3 months	SG versus CG: ↓ DFI after th.: 29.57 ± 12.19 versus 34.09 ± 10.32, *P* < 0.05		

Ménézo et al. [43]	2007	At least TWO pervious failures IVF or ICSI, DFI >15%/DFI and the degree of sperm decondensation (SCSA)	58	Vitamins C and E 400 mg each, *β*-carotene 18 mg, Zn 500 *μ*mol, and Se 1 *μ*mol/90 days	↓DNA fragmentation: −9.1%, *P* < 0.0004		↑ in sperm decondensation (+22.8%, *P* < 0.0009).

Greco et al. [44]	2005	TUNEL >15%/basic sperm parameters, TUNEL	38 (26 OAT + 6 OT + 6 normal)	Vit. C 1 g + vit. E 1 g/2 months	TUNEL positive sperm: 24.0 ± 7.9 to 8.2 ± 4.3 (*P* < 0.001) Clinical pregnancy after ICSI: from 6.9% to 48.2% (*P* < 0.05) Implantation rate after ICSI: from 2.2% to 19.6% (*P* < 0.01)	Sperm conc: 17.9 ± 16.3 to 18.3 ± 17.9 Sperm motil.: 40.6 ± 24.8 to 39.9 ± 19.0 Normal sperm morph.: 10.5 ± 8.3 to 9.6 ± 0.4, all *P* > 0.05	

Greco et al. [45]	2005	TUNEL >15%/basic sperm parameters, TUNEL	SG: 32 PG: 32	Vit. C 1 g + vit. E 1 g/2 months	SG: ↓ fragm. DNA: 22.1 ± 7.7 → 9.1 ± 7.2 (*P* < 0.001)	PG: TUNEL: 22.4 ± 7.8 → 22.9 ± 7.9	

Raigani et al. [46]	2013	OAT/sperm quality, sperm mitochondrial function, sperm chromatin status, semen and blood folate, zinc, B12, TAC, and MDA concentr.	83	Folic acid 5 mg/day ± Zn sulphate 220 mg/day versus placebo/16 weeks	Sperm chromatin integrity (%) ↑ in group receiving only Zn sulphate treatment (*P* = 0.048)	Sperm conc. ↑ in group receiving the combined th. of folic acid and Zn sulphate and also in the group receiving only folic acid th.; (*P* = 0.056 and *P* = 0.05, respectively).	

Tremellen et al. [47]	2007	Male factor infertility, TUNEL >25%/embryo quality, pregnancy and fertilization rate after IVF-ICSI	SG: 36 PG: 16	Menevit (likopen, vit. C, vit. E, Zn, Se, folate, and garlic)/3 months	Pregnancy rate after ICSI in SG: 38.5%, versus PG: 16% (*P* = 0.046)		

Safarinejad et al. [48]	2011	iOAT/semen parameters and TAC of seminal plasma	260 (SG: 130, PG: 130)	Saffron 60 mg/day/26 weeks		No statistically significant improvements in either group in any of the studied semen parameters	

Nadjarzadeh et al. [49]	2011	iOAT/basic sperm parameters, TAC	47	CoQ10 200 mg/day/12 weeks versus placebo	SG: ↑ TAC (*P* < 0.05)	Semen parameters of CoQ10 group	

Comhaire et al. [50]	2000	Infertile men/sperm characteristics, ROS, fatty acids of sperm membrane phospholipids, sperm oxidized DNA (8-OH-dG), and induced AR	27	N-acetyl-cysteine or vitamins A + E and essential fatty acids	↓ ROS, ↑ AR	No improvement in sperm motility and morphology or ↓ of round cells and white blood cells in semen. Sperm concentration ↑ in oligozoosp. men (7.4 ± 1.3 to 12.5 ± 1.9 million/mL).	

Legend: Addyzoa: Gokshura (*Tribulus terrestris*) 200 mg, Ashtavarga 200 mg, Guduchi (*Tinospora cordifolia*) 150 mg, Ashwagandha (*Withania Somnifera*) 150 mg, Amalaki (*Emblica officinalis*) 75 mg, Balamool (*Sida cordifolia*) 75 mg, Vridhadharu (*Argyreia speciosa*) 75 mg, Shatavari (*Asparagus racemosus*) 75 mg, Shwet musli (*Chlorophytum arundinaceum*) 150 mg, Shuddha kapikachchhu (Purified *Mucuna pruriens*) 150 mg, Varahikand (*Tacca aspera*) 30 mg, Chopchin (*Smilax china*) 30 mg, Vidarikand (*Ipomoea digitata*) 30 mg, Munjatak (*Eulophia campestris*) 15 mg, Purnachandrodaya rasa 45 mg, Suvarnavang 30 mg, Muktashukti bhasma 30 mg, Suvarnamakshik bhasma 30 mg, Shilajit shuddha 30 mg, Abhrak bhasma 15 mg, Makardhwaj rasa 15 mg, Rasa sindur 5 mg; AR: acrosome reaction; CG: control group; DDS: DNA degraded sperm; DFI: DNA fragmentation index; Fertilovit Mplus: L-citrulline (20.2 %), L-carnitine-L-tartrate, D-alpha-tocopheryl acetate, hydroxypropyl methylcellulose (capsule coating), acidifier tartaric acid, L-ascorbic acid (6.7%), parting compound silicon dioxide, calcium carbonate, lycopene, N-acetyl-L-cysteine, glutathione (reduced), corn starch, zinc oxide, coenzyme Q10, vegetable oil, shellac coating, pteroyl-L-glutamate, sodium selenite, coloring agent titanium dioxide (capsule), coloring agent orange yellow S (capsule); CoQ10: coencyme Q10, FISH: fluorescent in situ hybridization; FSH: follicle-stimulating hormone; ICSI: intracytoplasmic sperm injection; iOAT: idiopathic OAT; IVF: in vitro fertilization; LH: luteinizing hormone; MDA: malondialdehyde; MSOME: motile sperm organelle morphology examination; OAT: oligoasthenoteratozoospermia; PG: placebo group; ROS: reactive oxygen species; Sairei-to: a Chinese herbal drug; SCSA: sperm chromatin structure assay; Se: selenium; SG: study group; T: testosterone; TAC: total antioxidant capacity; TUNEL: TdT (terminal deoxyribonucleotidyl transferase)—mediated dUTP nick-end labeling; Xuanju: *Formica fusca*, Herba epimedii, Fructus cnidii, and *Fructus lycii*; Zn: zinc.
